# Allogeneic hematopoietic stem cell transplantation outcome in oldest known surviving patients with Wiskott-Aldrich syndrome

**DOI:** 10.1016/j.jacig.2023.100191

**Published:** 2023-11-22

**Authors:** Ariharan Anantharachagan, Sook Yin Loh, Siobhan O. Burns, Arian Laurence, Susan Tadros, Eleni Tholouli, Yadanar Lwin, Nicolas Martinez-Calle, P. Vaitla, Emma C. Morris

**Affiliations:** a Department of Allergy and Clinical Immunology, Lancashire Teaching Hospitals NHS Foundation Trust, Preston, United Kingdom; b Department of Immunology, Lancashire Teaching Hospitals NHS Foundation Trust, Preston, United Kingdom; c University College London Institute of Immunity and Transplantation, London, United Kingdom; d Department of Immunology, The Royal Free London NHS Foundation Trust, London, United Kingdom; e Department of Clinical Haematology, University College London NHS Foundation Trust, London, United Kingdom; f Manchester University NHS Foundation Trust, Department of Haematology, Manchester, United Kingdom; g Department of Haematology, Nottingham, United Kingdom; h Department of Immunology Nottingham University Hospitals, NHS Trust, Nottingham, United Kingdom

**Keywords:** Wiskott-Aldrich syndrome, WAS, hematopoietic stem cell transplantation, HSCT, adult

## Abstract

Regardless of their age, adult patients with Wiskott-Aldrich syndrome should be considered for hematopoietic stem cell transplantation if clinically indicated.

Wiskott-Aldrich syndrome (WAS) is a rare, X-linked combined immune deficiency caused by mutations in the *WAS* gene.^1^ Its clinical features include eczema, thrombocytopenia, life-threatening infections, autoimmunity, and malignancy.^1^^,^^2^ The median survival of patients who lack the Wiskott-Aldrich syndrome protein (WASP) expression and have not undergone hematopoietic stem cell transplantation (HSCT) is less than 20 years.^3^ Until recently, the potentially curative treatments, allogeneic HSCT and gene therapy, have been reserved for children owing to the risks of higher transplant-related morbidity and mortality in adults with significant WAS-related complications before HSCT.^1^

Here, we present the 2 oldest patients with WAS reported to date who have undergone HSCT. Both patients received fludarabine, treosulfan, and thiotepa (FTT)-conditioned matched unrelated donor (MUD) allografts (9 of 10 and 10 of 10 HLA-matched, respectively). They underwent *in vivo* T-cell depletion with alemtuzumab. Cyclosporine and mycophenolate mofetil were used for graft-versus-host disease (GVHD) prophylaxis and letermovir was used for cytomegalovirus (CMV) prophylaxis.

## Patient 1

A 44-year-old male was clinically diagnosed with WAS shortly after birth. He had 2 affected brothers, and his mother was a known *WAS* gene carrier. One of his brothers died at age 4 years following an intracranial haemorrhage, and the other died at age 44 years with complications of large vessel vasculitis. Subsequent genetic testing identified hemizygosity for the c.257G>A (p.Arg86Pro), a known pathogenic variant in the *WAS* gene associated with reduced WASP expression (4.5%) by flow cytometry of peripheral blood.

During childhood he had mild eczema, profound thrombocytopenia (platelet count < 10 × 10^9^/L), and recurrent sinusitis. By adulthood he had recurrent sinusitis and ear infections, with occasional chest infections. At age 36 years he developed a subdural haematoma that required surgical evacuation with blood product support. He commenced IRT at age 38 years following pneumonia and documentation of failed test vaccinations. Before starting IRT, his total IgG and IgA levels were normal, with a reduced IgM level (0.29 g/L). Chest imaging demonstrated moderate bronchiectasis. He had no history of autoimmunity or lymphoproliferation. He was 42 years old when referred for consideration of HSCT shortly after the death of his older brother owing to complications of a large vessel vasculitis. His WAS score (defined in Albert et al^4^) before HSCT was 4.

He underwent HSCT at age 43 years (FTT alemtuzumab [60 mg], 10 of 10 MUD allograft, CMV +/+, *Toxoplasma* –/–, hepatitis B virus –/–, and blood group A Rhesus factor and D-antigen [RhD]-positive/blood group O RhD-positive). At last follow-up, 18 months after HSCT, he had trilineage 100% donor chimerism and was no longer undergoing systemic immune suppression and IRT, with only minimal skin GVHD. His full blood count and white cell differential, including platelets, were normal. His times to platelet engraftment >20 × 10^9^/L and >50 × 10^9^/L were 7 and 34 days, respectively. His times to neutrophil engraftment >0.5 × 10^9^/L and >1.0 × 10^9^/L were 14 and 16 days, respectively. His early post-HSCT complications included adenoviremia requiring cidofovir, BK viruria, chronic renal impairment, and acute GVHD of the skin (grade 2). At last follow-up, his renal function was normal. He developed mild coronavirus disease 2019 (COVID-19) at 12.5 months after HSCT and was treated with nirmatrelvir with ritonavir (Paxlovid) with complete resolution of symptoms. IRT was stopped at 10 months after HSCT with no subsequent bacterial infections to date. Administration of cyclosporine was stopped at 13 months after HSCT. Test vaccination responses (14 months after HSCT) to 1 dose of Prevenar 13 (Pneumococcal 13-valent conjugate vaccine [diphtheria CRM197 Protein] vaccine) and Menitorix (*Haemophilus influenzae* type b [Hib]/ Meningococcal C [MenC] vaccine) identified protective antibodies to 2 of 9 pneumococcal serotypes present in Prevenar 13 (normal response is ≥0.35 μg/mL to ≥50% serotypes tested) and adequate antibodies to *Haemophilus influenzae* type B (≥0.15 mg/L) at 0.22 mg/L.

## Patient 2

A 41-year-old male presented with a purpuric rash and thrombocytopenia at age 3 months. There was a positive family history, with 2 maternal uncles having died of WAS-related complications at a young age. Genetic testing identified hemizygosity for C.763delC (p.Gln255ArgfsX6) , a pathogenic variant in the *WAS* gene, and his WASP expression by flow cytometry was low (10.4%), which is consistent with WAS. In early childhood he developed moderate eczema and had recurrent ear, nose, and throat infections. Thrombocytopenia resulted in several major epistaxes and 2 instances of hemarthroses of the right knee following an osteochondral fracture. In adulthood he developed mild bronchiectasis and inflammatory arthropathy, and he had recalcitrant human papillomavirus warts affecting his hands. He remained stable with conservative management (co-trimoxazole, aciclovir, IRT, and tranexamic acid) for several years.

At age 38 years he was diagnosed with diffuse large B-cell lymphoma (Epstein-Barr early RNA–negative) and received 6 cycles of rituximab, cyclophosphamide, hydroxydaunorubicin, oncovin, and prednisone chemotherapy with additional romiplostim for platelet support, achieving complete remission according to positron emission tomography–computed tomography. Following chemotherapy, he was assessed for HSCT.

He underwent HSCT at age 39 years (FTT alemtuzumab [60 mg], 9 of 10 [A mismatch] MUD allograft, CMV +/+, *Toxoplasma* –/–, and blood group A RhD-positive/blood group AB RhD-negative). At 18 months follow-up, he had trilineage 100% donor chimerism but continued receiving cyclosporine for chronic GVHD of the gut. His full blood count and white cell differential, including platelets, were normal. His times to platelet engraftment >20 × 10^9^/L and >50 × 10^9^/L were 12 and 16 days, respectively. His times to neutrophil engraftment >0.5 × 10^9^/L and >1.0 × 10^9^/L were 13 and 22 days, respectively. His early post-HSCT course was complicated by 4 episodes of herpes simplex virus 2 requiring aciclovir, foscarnet, and topical cidofovir; grade 2 acute GVHD of the skin and gut; and adenoviremia requiring cidofovir. At 6 months after HSCT he developed Lhermitte sign (an electric shock–like sensation on neck flexion) and clinical features of a progressive axonal neuropathy (distal muscle wasting and weakness). Electromyography confirmed axonal neuropathy. Magnetic resonance imaging and cerebrospinal fluid examination have not identified an underlying cause. At last follow-up, the patient’s Lhermitte sign was resolving. He developed asymptomatic COVID-19 at 8 months after HSCT, which was treated with sotrovimab, and a further episode 8 months later, which improved after 3 days of remdesivir. Assessment of test vaccine responses are pending after cessation of cyclosporine and IRT.

Recent data have demonstrated that good outcomes can be achieved for adults who undergo HSCT for combined immune deficiencies.^5^^,^^6^ The aforementioned cases show that HSCT is feasible in older patients with WAS and should be considered for those for whom a donor is available. In these 2 patients, longer follow-up is required to provide evidence of definitive improvement in quality of life and disease-free survival.

## Disclosure statement

Supported by the 10.13039/501100000272National Institute for Health Research University College London Hospitals Biomedical Research Centre (to E.C.M.).

Disclosure of potential conflict of interest: A. Anantharachagan has received honoraria from Takeda (for chairing a webinar and advisory board work) and support for attending a conference. S. Tadros has received support from CSL Behring for attending a conference. E. Morris has received salary support from the 10.13039/501100000765University College London Hospitals 10.13039/501100000272National Institute for Health Research Biomedical Research Centre and support from GSK, AstraZeneca, GE Healthcare, and Orchard Therapeutics; in addition, she is an Orchard Therapeutics Limited data and safety and monitoring board member for the X-Linked Chronic Granulomatous Disease Phase I trial, a panel member on the Wellcome Trust Career Development Panel, and a scientific founder of Quell Therapeutics Limited. The rest of the authors declare that they have no relevant conflicts of interests.
